# The relevance of the psychological evaluation in drug dependence

**Published:** 2014

**Authors:** G Popescu, C Negrei, D Bălălău, AM Ciobanu, D Baconi

**Affiliations:** * Department of Toxicology, “Carol Davila” University of Medicine and Pharmacy, Faculty of Pharmacy, Bucharest, Romania; ** Department of Drug Control, “Carol Davila” University of Medicine and Pharmacy, Faculty of Pharmacy, Bucharest, Romania

**Keywords:** methadone, drug dependence, drug dependence

## Abstract

Psychological interventions are considered a central part of the individual psychotherapy in the rehabilitation counseling of psychiatrically symptomatic drug-dependent patients during methadone maintenance treatment in community programs. The need for psychological counseling should be evaluated for each individual patient.

Medication is an important part of the treatment and individual psychotherapy focuses on the reduction or total cessation of drug use.

The Recipient is G.M. 31, sentenced to a seven-year term of imprisonment for trafficking and use of and high-risk drugs, diagnosed on admission with opioid and methadone dependence, withdrawal syndrome.

Following the observation and psychological evaluation, psychiatric and clinical examination, initiation of methadone substitution treatment was recommended, according to the following regimen: twelve 2.5 mg tablets for the first 2 days, followed by increase with about 5 mg per week until the complete remission of withdrawal symptoms, stabilization of the dose but not exceeding 200 mg methadone hydrochloride per day.

Specialist monitoring, specialized counseling and individual and group psychotherapy were provided.

## Introduction

In most programs, psychological interventions are considered a central part of methadone treatment [**1**].

Various researches have revealed certain program characteristics associated with successful treatment, such as comprehensive services and integration of health and administrative services as well as psychological counseling.

Researchers have studied whether or not patients who had received counseling and other psychosocial services during treatment had better outcomes than those strictly on methadone therapy only [**2**-**4**].

The importance of psychological counseling as an additional component to methadone treatment is widely accepted. However, limitations to counseling have to be mentioned.

As with any patient receiving any kind of treatment, wherever therapy was administered, individual treatment of patients under methadone maintenance may have different needs and respective responses to treatment may vary as well.

The need for psychological counseling should be evaluated for each individual patient, as there are patients requiring an extended assistance in order to restore order in their lives, and therefore counseling is needed to a greater extent [**5**].

On the other hand, there is no reason for stable patients, with major life problems, to be provided counseling in excess.

Psychological techniques have become a core element of good clinical practice against opioid dependence and they are important adjuncts to pharmacotherapy in most countries.

Clinical psychology provides models for drug addiction, combining social and neurobiological theories.

For instance, the motivational interview is important both for the assessment process for patient placement in therapy, and prevention of relapse during the detoxification treatment. Patients with concurrent mental illnesses may benefit from targeted therapies such as cognitive behavioral therapy [**6**-**8**].

Cognitive-behavioral techniques, including individual or group psychological counseling are essential components of addiction treatment.

While in therapy, patients strengthen their motivation, acquire skills supporting them to resist the temptation of use, encounter gratifying activities not involving drugs able to replace the former ones, and improve their problem solving skills.

Behavior therapy facilitates interpersonal relationships and individual availability to operate at family and community levels.

Medication is an important part of treatment, particularly when accompanied by counseling and other behavioral therapies.

Individual psychotherapy focuses on reduction or total cessation of drug use, envisaging other aspects of the drug-addicted person’s life not working properly, such as their inability to keep their job, involvement in illegal activities, family neglecting.

Focusing on the achievement of changes in the addictive behavior, individual therapy provides the patient with the knowledge and skills able to help them achieve and maintain abstinence [**9**,**10**].

## Case report

On admission, the patient to be placed in the methadone program undergoes:

- Assessment at the reception point reception conducted by the multidisciplinary team (physician, psychologist) for the establishment of the degree of motivation for replacement therapy initiation;

- Positive urine test for opioids on admission – mandatory prerequisite for inclusion in the treatment;

- Signing of the consent to treatment - agreement with medical, psychological and social assistance;

- Assessment of the current clinical condition.

The Recipient is G.M., 31 years old, sentenced to a seven years term of imprisonment for trafficking and use of and high-risk drugs, diagnosed on admission with opioid and methadone dependence, withdrawal syndrome. 

The following were revealed after the application of the toxophilic sheet: 

• drug user since 1993, 

• history of overdose in 2007; 

• polyvalent drug user: 

• inhibitors: frequent heroin, methadone use; codeine and buprenorphine, occasionally; 

• stimulants: frequent use of cocaine, methamphetamine and ecstasy; speed-ball, occasionally;

• hallucinogens: frequent consumption of Joints and blunts (cannabis, hashish, marijuana) and ketamine; LSD, occasionally;

• anxiolytics: diazepam, nitrazepam, lorazepam, fluorazepam, occasionally, but in large quantities (ca.10-15 tablets) together with the heroin dose;

• hypnotics: dormital, ciclobarbital, Phenobarbital, occasionally; mushrooms and ethers, occasionally; 

• mixtures of alcohol and drugs, alcohol and tablets, drugs and tablets, alcohol, drugs and tablets;

Following the observation and psychological evaluation, the following conclusions were revealed: 

• appropriate contact psychic; 

• adequate sensory - perceptual development;

• fine and gross motor skills within normal parameters; 

• average cognitive level, appropriate knowledge base;

• satisfactory operational functions without quantitative or qualitative disorders at assessment time; 

• short- and long- term memory within the normal parameters; 

• amnesic efficiency; 

• temporo-spatially oriented, spontaneous and distributive attention in place, appropriate eye contact; 

• positive affective tone, slight tendency towards the simulation of emotional behavior, adequate dispositional state, slight relational immaturity; 

• partly reproductive voluntary imagination type, appropriate developmental stage; 

• predominantly extrinsic, selective motivation, unrealistic decision - motivational balance; 

• slow reaction, withdrawn, average adaptability, passive-aggressive, predominantly sanguine temperament, predominantly extroverted personality; 

• voluntary control in place, minimal voluntary effort; 

• optimistic, appropriate attitude behavior approving; 

• literary inclinations; psychosocial immaturity, personality with elements of antisocial behavior.

In result of the application of the motivational interview, the subject was revealed to be in the preparation stage, combining intentional and behavioral criteria; the patient intends to change in the near future and has already made minor changes, however, not yet meeting the criteria for the action stage, i.e. long-term abstinence (minimum 3 months) has not been achieved yet.

On psychiatric examination, the patient complained of the following: 

- withdrawal symptoms: insomnia, chills, sweating, feeling cold, musculoskeletal, abdominal pain, rhinorrhea, nausea, vomiting, diarrhea; 

- overall careless looks, immobile facial expression, focused gaze, cooperative attitude, proper psychic contact, auto- and allo-psychologically oriented;

- spontaneous and distributive attention, short- and long- term memory within normal parameters;

- average cognitive level without quantitative or qualitative changes in the sphere of thought; 

- prevailingly impulsive, irritable, impulsive, demanding dispositional tone, diminished psychomotor agitation.

Heroin, methadone and benzodiazepines tests were positive.

The initiation of methadone substitution treatment was recommended, according to the following regimen: twelve 2.5 mg tablets for the first 2 days, followed by an increase with about 5 mg per week until the complete remission of withdrawal symptoms, stabilization of the dose but not exceeding 200 mg methadone hydrochloride per day.

Additionally, specialist monitoring, specialized counseling and individual and group psychotherapy were provided.

## Discussion

Following the corroboration of overall investigations established on admission, the patient was recommended for inclusion in a therapeutic module for drug users. Behavioral therapies can be used to make effective pharmacotherapies available to a wider proportion of substance abusers being a point that has considerable implications beyond making methadone a more viable treatment option.
